# Locoregional and Distant Recurrence Patterns in Young versus Elderly Women Treated for Breast Cancer

**DOI:** 10.1155/2015/213123

**Published:** 2015-04-09

**Authors:** Soumon Rudra, David S. Yu, Esther S. Yu, Jeffrey M. Switchenko, Donna Mister, Mylin A. Torres

**Affiliations:** Department of Radiation Oncology, Winship Cancer Institute, Emory University School of Medicine, Atlanta, GA 30322, USA

## Abstract

*Objective*. This study examined recurrence patterns in breast cancer patients younger than age of 40 and older than age of 75, two groups that are underrepresented in clinical trials and not routinely screened by mammography. *Methods*. The records of 230 breast cancer patients (*n* = 125 less than 40 and *n* = 105 greater than 75) who presented to the Emory University Department of Radiation Oncology for curative treatment between 1997 and 2010 were reviewed. Data recorded included disease presentation, treatment, and areas of locoregional recurrence. *Results*. Women less than 40 years of age had higher rates of locoregional recurrence (20% versus 7%, *P* = 0.004) and distant recurrence (18% versus 5%, *P* = 0.003) than patients above 75 years of age. On multivariate analysis, patient age less than 40 was the only significant predictor of locoregional recurrence (*P* = 0.018). In a univariate analysis of each age group, receptor status and postlumpectomy radiation were significant predictors of locoregional recurrence-free survival in younger women while mammography screening predicted for distant recurrence-free survival in older patients. *Conclusion*. The factors identified in our age-stratified analysis highlight patients who are at high risk of locoregional and distant recurrence. Future studies aimed at enhancing therapies in young patients are warranted.

## 1. Introduction

Few studies have compared locoregional recurrence (LRR) and distant recurrence (DR) outcomes in women less than 40 with those above 75 years: two cohorts of women that are underrepresented in randomized trials of breast cancer treatments. Furthermore, these same groups of women fall outside breast cancer screening guidelines likely leading to underdetection of disease. Current mammography recommendations from the American Cancer Society (ACS) initiate screening at age of 40, and the US Preventive Task Force (USPTF) states that there is insufficient evidence to support screening mammograms in older women, particularly those above the age of 75. However, patients under the age of 40 are more likely to be diagnosed with advanced stage disease and die more often due to their breast cancer [1]. Breast cancer in older women is generally thought to be relatively indolent [2], but some studies suggest that even older women may present with late-stage disease [3] and have poorer disease specific survival due to a lack of routine mammography screening [4]. Moreover, older women, unlike their younger counterparts, may have significant comorbidities that preclude standard therapeutic options and consequently adversely affect breast cancer specific outcomes.

LRR is correlated with increased risk for DR and poor survival outcomes [5]. One of the primary purposes of radiation following surgery is to reduce LRR rates and improve breast cancer specific survival and reduce the number of secondary surgeries and treatments. LRRs can occur in different tissue sites including the ipsilateral breast, chest wall, axillary, supraclavicular, and internal mammary lymph nodes. A local recurrence within the breast may require a complete mastectomy, as salvage treatment, while a chest wall recurrence may need excisional surgery along with radiation with or without systemic therapy [6]. Preventing a LRR is an important factor driving improvements in the treatment of primary breast tumors, but current guidelines for deciding lumpectomy versus mastectomy are based on potential cosmetic outcome, history of collagen vascular diseases and prior radiation, and patient and physician preference. The decision to treat with radiation is largely driven by type of surgery and presence of lymph node disease and positive margins. Whether to include the regional lymph nodes in the radiation fields is based on extent of lymph node involvement and initial size of the primary tumor. Few studies have examined whether young versus older patients have different patterns of LRR and whether patient age should be taken into account when determining surgery type and radiation treatment and field design.

Identifying risk factors contributing to locoregional recurrence-free survival (LRFS) and distant recurrence-free survival (DRFS) can help clinicians decide on appropriate treatments for patients in these age groups. Due to the importance of locoregional control in overall breast cancer prognosis, the aim of this study was to evaluate patterns and risk factors for LRFS and DRFS in patients younger than 40 and women older than 75, two understudied populations who are historically underrepresented in clinical trials and fall out of the range of screening guidelines.

## 2. Materials and Methods

The medical records of women evaluated for their breast cancer in Emory University Hospital's Department of Radiation Oncology from 1997 until 2010 were reviewed. Exclusion criteria included patient age between 40 and 74 years and stage IV or inflammatory breast cancer. In addition, among patients treated with chemotherapy, those who did not receive standard anthracycline or taxane-based treatments were excluded. In total, 230 women met eligibility criteria for this study with 125 patients below the age of 40 and 105 subjects above the age of 75.

Tumor receptor status was determined by immunohistochemistry. Her-2-neu status was recorded as positive for tumors that stained 3+ on immunohistochemistry. For Her-2-neu tumors that were 2+, confirmatory fluorescence in situ hybridization (FISH) testing was performed. Tumors were staged according to the 2010 American Joint Committee on Cancer (AJCC) guidelines.

Outcomes included LRFS, DRFS, and overall survival (OS). LRR was defined as a biopsy proven recurrence of the primary breast cancer within the ipsilateral breast, chest wall, axillary, internal mammary, or supraclavicular lymph nodes. DR was defined as a biopsy proven recurrence in any other location of the body. Descriptive statistics were generated for all variables, summarized with frequencies and percentages. Covariates as well as predictors of LRFS, DRFS, and OS were compared across age groups using chi-squared tests or Fisher's exact tests, where appropriate. Univariate (UV) Cox proportional hazards models were fit for the outcomes listed above, using age as the primary predictor. Multivariate (MV) Cox models were fit for overall LRFS, DRFS, and OS. In addition, survival curves were generated for each outcome using the Kaplan-Meier method, stratified by age group. Outcomes such as 5-year survival rates were reported for each group, and differences in 5-year survival were compared using a *z*-test. Univariate analysis was performed to determine predictors of LRFS in each age cohort. Significance was assessed at the 0.05 level. Survival analysis was performed in SAS 9.3, and survival curves were generated in *R*. Firth's penalized maximum likelihood estimation was used in the survival models, in order to reduce bias in the parameter estimates and confidence intervals, as well as handle empty cells.

## 3. Results

### 3.1. Tumor Characteristics

The majority of younger women (88%) presented with cancers that were symptomatic while the majority of older women (63.1%) were more likely to have cancers detected by mammography (*P* < 0.001). Tumor grade and stage were also significantly different between the two groups. In the younger cohort, 55.9% of patients had grade 3 tumors compared to 32% of older patients (*P* = 0.001). Clinical stage at presentation (young versus old) was Stage 0/I (21.0% versus 65.4%, *P* < 0.001) and Stage II/III (79.0% versus 34.6%, *P* < 0.001). Additional information on tumor characteristics is available in Table 1.

### 3.2. Treatment Characteristics

A greater proportion of younger than older patients received chemotherapy. Chemotherapy was given in the neoadjuvant setting to 66.7% of younger patients versus 6.7% of older patients (*P* < 0.001). Chemotherapy was given in the adjuvant setting to 30.4% of younger patients and 9.52% of older patients (*P* < 0.001).

Lumpectomy was the most common surgical procedure in both groups of patients but the distribution of surgical procedures was significantly different between the age groups (*P* < 0.001). Surgical margins greater than 2 millimeters were achieved in 87.0% of younger women and in 77.5% of older women (*P* = 0.153). Approximately, 92.8% of younger patients versus 92.4% of older patients underwent postlumpectomy radiation, and 88.0% of younger patients versus 80.0% of older patients with lymph node positive disease underwent postmastectomy radiation. The differences were not statistically significant. Additional treatment characteristics are listed in Table 1.

### 3.3. Outcomes

**Figure 1:**
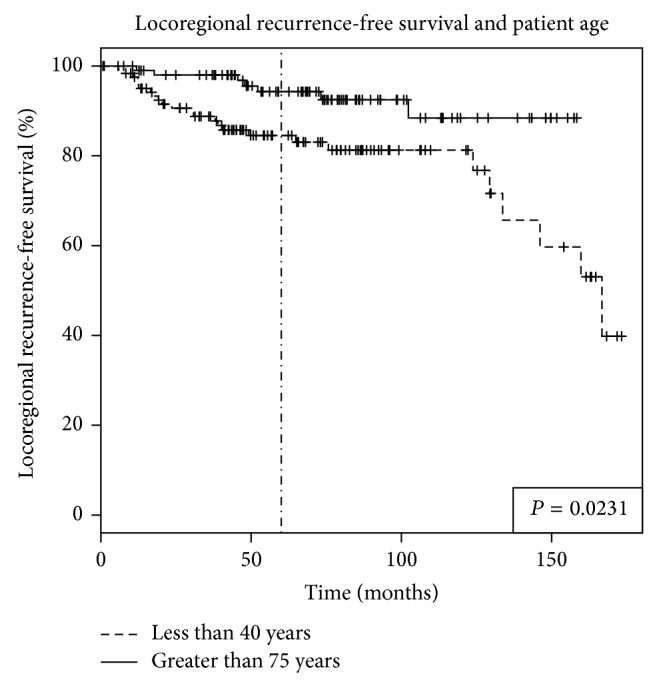
Locoregional recurrence-free survival (LRFS) in breast cancer patients based on patient age. Women younger than age of 40 had a significantly worse LRFS than those above the age of 75. The 5-year rate of LRFS was 84.5% versus 94.3% (*P* = 0.0231).

**Figure 2:**
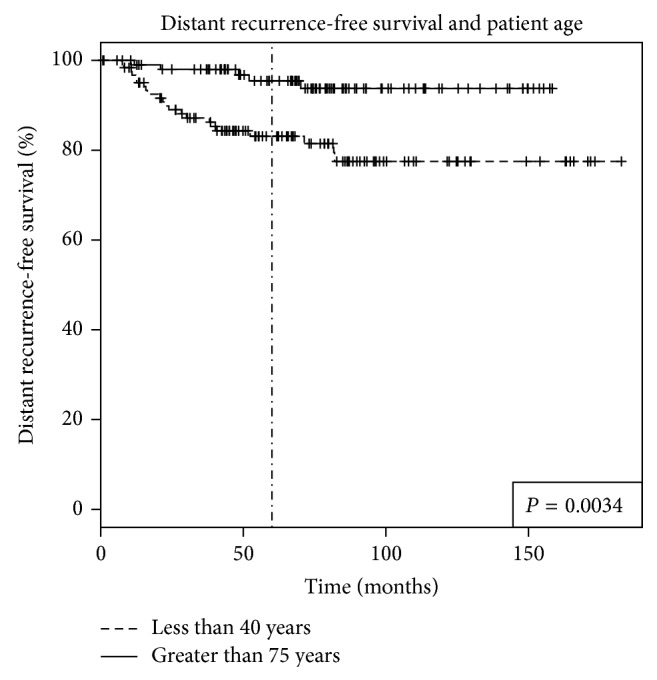
Distant recurrence-free survival (DRFS) in breast cancer patients based on patient age. Women younger than age of 40 had a significantly worse DRFS than those above the age of 75. The 5-year rate of DRFS was 83.1% versus 95.5% (*P* = 0.0034).

**Figure 3:**
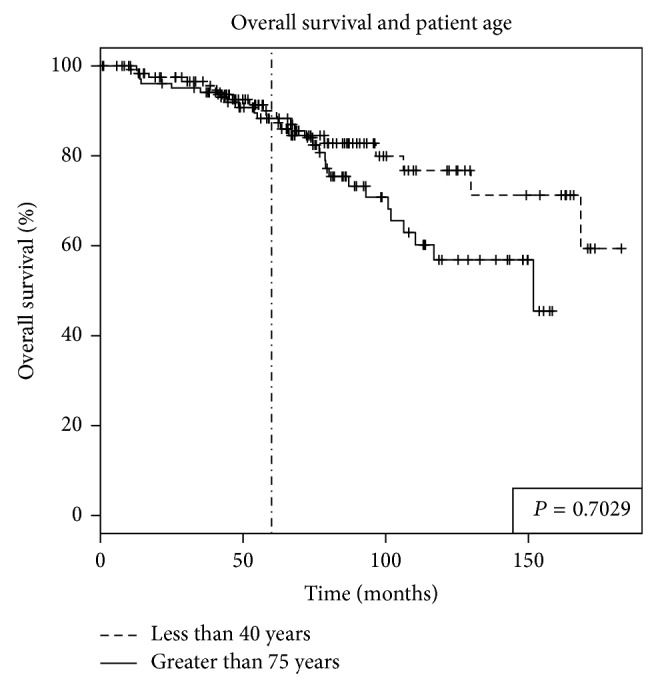
Overall survival (OS) in breast cancer patients based on patient age. Women younger than age of 40 and women older than age of 75 did not have significantly different OS. The 5-year rate of OS was 90% versus 88.3% (*P* = 0.7029).

The median follow-up period was 5.8 years (range from 1 month to 14.5 years) for both groups. LRFS rate was significantly lower in younger than older patients (84.5% versus 94.3%, *P* = 0.023) (see Figure 1). The DRFS rate was also significantly lower in younger women (83.1% versus 95.5%, *P* = 0.003) (see Figure 2). OS was not significantly different between younger and older women at 5 years (90% versus 88.3%, *P* = 0.703) (see Figure 3).

Age at diagnosis was associated with both LRFS and DRFS (HR: 3.1, 95% CI: (1.3, 7.2), *P* = 0.006; HR: 4.2, 95% CI: (1.6, 11.0), *P* = 0.002). Age remained significantly associated with LRFS in a multivariate model (*P* = 0.011). Grade was associated with overall survival in the UV model, while age was associated with overall survival in the MV model, adjusting for receptor status, grade, surgery type, and chemotherapy.

Among the 25 younger and 8 older patients who experienced a LRR, 64.0% of the younger patients recurred within breast/chest wall compared with 87.5% of older patients (*P* = 0.387). In addition, 44% of younger patients recurred within the draining lymphatics compared with 29% of older patients (*P* = 0.671). Among the younger patients with lymphatic recurrence, 36% had level I or II axillary involvement, 18% had supraclavicular node (SCN) involvement, 27% had both axillary and SCN involvement, and 18% had internal mammary node involvement. In older patients with a lymphatic recurrence, 50% had SCN involvement, 50% had axillary and SCN involvement, and none had internal mammary node involvement.

Univariate analysis was performed in each age group to determine if there were differences in predictors of LRFS and DRFS between groups. Among the younger patients, triple negative receptor status and lack of postlumpectomy radiation were significant predictors of lower LRFS (Table 2). Significant predictors for worse DRFS included mastectomy (as opposed to breast conserving surgery), use of adjuvant chemotherapy (significant log-rank *P* value and marginally significant hazard ratio *P* value), stage at diagnosis, nodal status, and use of postmastectomy radiation (Table 3). Among the older patients, there were no significant predictors for LRFS. Predictors of worse DRFS included type of surgery (i.e., mastectomy), use of neoadjuvant chemotherapy, lack of mammography screening (significant log-rank *P* value and marginally significant hazard ratio *P* value), and positive nodal status (significant log-rank *P* value and marginally significant hazard ratio *P* value) (Table 4). The small number of events in each age group precluded meaningful MV analysis.

## 4. Discussion

This study revealed that age at diagnosis is a strong predictor for LRFS and DRFS. Younger patients developed both types of recurrence more frequently than their older counterparts. Since these events carry poor prognoses for survival outcomes, it was necessary to identify appropriate risk factors in these understudied populations.

The tumor characteristics of our study are consistent with previous studies which indicate that younger patients tend to present with tumors that are self-detected rather than detected by screening mammography. Indeed, other studies have confirmed that young women with breast cancer are most likely to present with symptomatic, large palpable masses [1] in part due to lack of screening in this patient population. These tumors are generally poorly differentiated, higher grade and are associated with lymphovascular invasion [1–4]. Our results support these findings, as younger women presenting to our department for radiation tended to present with advanced stage disease as well. Furthermore, more advanced stage and higher grade tumors in young women have been linked with LRR in the literature [7].

Unlike the younger women in our study, the older women were diagnosed with smaller and lower grade tumors. Most of these tumors were discovered on screening mammography despite the lack of clear guidelines for this age group. In addition, studies of older women have generally reported favorable tumor characteristics [8, 9]. However, in one retrospective review of 135 women with a median age of 83 years, 59% of tumors were histological grade 2-3 with lymph node involvement [10]. In general, the favorable tumor characteristics in patients above 75 years of age contributed to lower LRR rates in our study.

Systemic chemotherapy and/or targeted agents were used on younger women more often than women above the age of 75 partly due to the higher stage at presentation. Systemic treatment recommendations for breast cancer are based on perceived benefits and potential toxicities such that decisions are often made based on age, cancer stage, patient performance status, and comorbidities. Fewer elderly women receive chemotherapy for their breast cancer [11, 12], and when they are prescribed systemic therapy, they are often given noncardiotoxic agents [13]. However, even with the use of chemotherapy, recurrence rates were significantly higher in the younger cohort due to the advanced stage at presentation.

In support of our findings, others have shown that breast cancer tends to recur at a higher rate in younger women [1, 3]. In an age-specific analysis of clinical outcomes, Anders et al. reported inferior disease-free survival (DFS) in women less than 45 as compared with those greater than 65 years, with the lowest DFS in women less than 40 years of age [14]. The younger patients in our study showed significantly worse LRFS and DRFS outcomes when compared with older patients, but only LRFS remained significantly associated with age in the MV model.

In an age-stratified analysis, we attempted to identify predictors for LRFS and DRFS in each age group. For younger patients, receptor status was associated with LRFS. In particular, triple negative receptor status had the strongest association with locoregional failure. Our study suggested that triple negative breast cancer (TNBC) patients should receive more aggressive treatment and surveillance recommendations in order to achieve better local control. Another predictor of worse LRFS in young patients was lack of postlumpectomy radiation. In a study by Dragun et al, TNBC patients who did not receive radiation had a higher risk of LRR despite lower staging at diagnosis [15]. After recognizing the high potential of LRR in triple negative cancers, the authors proposed strongly considering radiation in nontraditional patients such as “postmastectomy patients with <4 positive lymph nodes and/or tumors <5 cm in size” [15]. In our study, the younger patients also tended to recur in the regional nodal regions more than older patients. This data suggests that when young breast cancer patients are prescribed radiation, they may benefit from regional nodal radiation in addition to breast or chest wall treatment.

Among our patients, worse DRFS in young patients was predicted by multiple factors including stage at diagnosis, presence of nodal disease, mastectomy, use of adjuvant chemotherapy, and use of postmastectomy radiation. The worse outcomes associated with these breast cancer treatments are likely due to the initial advanced disease presentations of these patients. Given our results, future research should be devoted to improving screening techniques and guidelines to prevent advanced disease presentations in young patients.

The stratified analysis did not identify any risk factors for LRFS in the older cohort. However, worse DRFS was associated with positive nodal status, mastectomy, use of neoadjuvant chemotherapy, and lack of mammography screening. Although the older patients had significantly lower LRFS and DRFS rates, they did not have significantly better OS at 5 years. This is likely due to comorbidities unrelated to breast cancer. A recent SEER database study of approximately 64,000 women diagnosed with breast cancer at age of 66 and older found that elderly women with breast cancer were just as likely to die of cardiovascular disease as they were of their breast cancer [16]. The degree to which undertreatment impacts breast cancer outcomes in older women remains an area of current debate. Gajdos et al. compared 206 women whose ages are 70 and over and compared them with 920 younger women and found that 54% of the elderly group were undertreated; however, local and distant recurrence-free survival rates were comparable among the two cohorts of patients regardless of treatment [17]. These data strongly suggest that older women tend to die of non-breast cancer related causes in spite of less aggressive and nonstandard therapy for their breast cancer. Yet, one recent retrospective analysis of postmenopausal patients with early stage breast cancer who participated in the Tamoxifen Exemestane Adjuvant Multinational (TEAM) trial found that higher breast cancer-specific mortality with increasing age may be attributed to undertreatment [18]. In addition, Schonberg et al. report that patients who are 67 years or older with stage II or higher disease had increased mortality when compared with similarly aged patients without breast cancer [19]. While such studies call for aggressive standard treatment in older women, our study suggests that older women who are healthy enough candidates to be considered for radiation still tend to die of causes unrelated to their breast cancer irrespective of treatment.

The risk factors for locoregional and distant recurrences suggested by this study were developed through a retrospective method and the findings are therefore hypothesis generating. A prospective study must further evaluate these risk factors in order to establish the proper associations.

## 5. Conclusion

Women less than 40 years old suffer significantly worse LRFS and DRFS in spite of aggressive therapies. This is likely due to the combination of aggressive breast tumor characteristics and inadequate screening. In contrast, less aggressive treatment in older women with early stage tumors does not appear to affect their overall breast cancer outcomes. Future studies aimed at assessing biological risk factors for breast cancer are warranted independent of age. Such information could be used to more effectively detect breast cancer and potentially administer treatment in women not typically screened with mammography.

## Figures and Tables

**Table 1:** Breast cancer characteristics in younger and older women.

Characteristic	Age less than 40 *N* = 125 (%)^*^	Age greater than 75 *N* = 105 (%)^*^	*P* value

Receptor status			
ER/PR+^#^	71 (62.28)	73 (77.66)	0.036
Her2+	11 (9.65)	8 (8.51)	
Triple negative	32 (28.07)	13 (13.83)	
Grade			
1	14 (12.61)	26 (26)	0.001
2	35 (31.53)	42 (42)	
3	62 (55.86)	32 (32)	
Surgery type			
Lumpectomy	69 (55.2)	92 (87.62)	<0.001
Modified radical mastectomy	20 (16.0)	9 (8.57)	
Simple mastectomy	17 (13.6)	4 (3.81)	
Bilateral mastectomies	19 (15.2)	0 (0)	
Adjuvant chemotherapy			
No	87 (69.6)	95 (90.48)	<0.001
Yes	38 (30.4)	10 (9.52)	
Neoadjuvant chemotherapy			
No	41 (32.8)	98 (93.33)	<0.001
Yes	84 (67.2)	7 (6.67)	
Detected on mammography			
No	103 (88.03)	38 (36.89)	<0.001
Yes	14 (11.97)	65 (63.11)	
Stage at diagnosis			
0/I	25 (21.01)	68 (65.38)	<0.001
II/III	94 (78.99)	36 (34.62)	
Postmastectomy radiation			
No	16 (28.57)	3 (23.08)	1.000
Yes	40 (71.43)	10 (76.92)	
Postlumpectomy radiation			
No	5 (7.25)	7 (7.61)	0.931
Yes	64 (92.75)	85 (92.39)	
Final margin status			
Positive	2 (1.63)	2 (1.96)	0.153
Less than 2 mm	14 (11.38)	21 (20.59)	
Greater than 2 mm	107 (86.99)	79 (77.45)	

^*^Total patients for each characteristic may vary due to incomplete medical records for some patients.

^#^ER/PR+: estrogen receptor/progesterone receptor positive.

**Table 2:** Univariate analysis of risk factors for locoregional free survival in younger women.

Characteristic	*N* (%)^*^	Hazard ratio (95% CI)	Hazard ratio *P* value	Log-rank *P* value

Receptor status				
ER/PR+^#^	71 (62.28)	0.35 (0.15–0.85)	0.021	0.033
Her2+	11 (9.65)	0.34 (0.06–1.99)	0.231	
Triple negative	32 (28.07)	—		
Grade				
1	14 (12.61)	1.11 (0.36–3.42)	0.854	0.949
2	35 (31.53)	0.90 (0.34–2.40)	0.832	
3	62 (55.86)	—		
Surgery type				
Lumpectomy	69 (55.2)	1.20 (0.52–2.73)	0.672	0.634
Mastectomy	56 (44.8)	—		
Adjuvant chemotherapy				
No	87 (69.6)	0.87 (0.38–1.99)	0.736	0.781
Yes	38 (30.4)	—		
Neoadjuvant chemotherapy				
No	41 (32.8)	1.83 (0.82–4.07)	0.137	0.134
Yes	84 (67.2)	—		
Detected on mammography				
No	103 (88.03)	0.40 (0.15–1.05)	0.062	0.075
Yes	14 (11.97)	—		
Stage at diagnosis				
0/I	25 (21.01)	2.02 (0.85–4.79)	0.111	0.120
II/III	94 (78.99)	—		
Nodal status				
Positive	41 (32.8)	0.78 (0.33–1.85)	0.572	0.512
Negative	84 (67.2)	—		
Postmastectomy radiation				
No	16 (28.57)	1.18 (0.30–4.66)	0.816	0.901
Yes	40 (71.43)	—		
Postlumpectomy radiation				
No	5 (7.25)	6.34 (1.46–27.55)	0.014	0.017
Yes	64 (92.75)	—		
Final margin status				
Positive	(1.63)	1.92 (0.10–36.83)	0.665	0.774
Less than 2 mm	14 (11.38)	1.55 (0.47–5.09)	0.474	
Greater than 2 mm	107 (86.99)	—		

^*^Total patients for each characteristic may vary due to incomplete medical records for some patients.

^#^ER/PR+: estrogen receptor/progesterone receptor positive.

**Table 3:** Univariate analysis of risk factors for distant recurrence-free survival in younger women.

Characteristic	*N* ^*^	Hazard ratio (95% CI)	HR *P* value	Log-rank *P* value
Receptor status				
ER/PR+^#^	71 (62.28)	0.87 (0.33–2.31)	0.786	0.822
Her2+	11 (9.65)	0.71 (0.11–4.48)	0.716	
Triple negative	32 (28.07)	—		
Grade				
1	14 (12.61)	0.80 (0.19–3.36)	0.766	0.818
2	35 (31.53)	0.82 (0.28–2.35)	0.706	
3	62 (55.86)	—		
Surgery type				
Lumpectomy	69 (55.2)	0.28 (0.11–0.72)	0.008	0.003
Mastectomy	56 (44.8)	—		
Adjuvant chemotherapy				
No	87 (69.6)	0.40 (0.17–0.93)	0.033	0.027
Yes	38 (30.4)	—		
Neoadjuvant chemotherapy				
No	41 (32.8)	0.89 (0.35–2.26)	0.812	0.735
Yes	84 (67.2)	—		
Detected on mammography				
No	103 (88.03)	1.10 (0.29–4.25)	0.888	0.692
	14 (11.97)	—		
Stage at diagnosis				
0/I	25 (21.01)	0.09 (0.00–1.55)	0.097	0.018
II/III	94 (78.99)	—		
Nodal status				
Positive	41 (32.8)	2.58 (1.12–5.98)	0.027	0.020
Negative	84 (67.2)	—		
Postmastectomy radiation				
No	16 (28.57)	0.17 (0.03–0.98)	0.048	0.014
Yes	40 (71.43)	—		
Postlumpectomy radiation				
No	5 (7.25)	1.19 (0.05–26.66)	0.912	0.534
Yes	64 (92.75)	—		
Final margin status				
Positive	2 (1.63)	1.47 (0.08–27.41)	0.795	0.771
Less than 2 mm	14 (11.38)	0.88 (0.23–3.41)	0.851	
Greater than 2 mm	107 (86.99)	—		

^*^Total patients for each characteristic may vary due to incomplete medical records for some patients.

^#^ER/PR+: estrogen receptor/progesterone receptor positive.

**Table 4:** Univariate analysis of risk factors for distant recurrence-free survival in older women.

Characteristic	*N* ^*^	Hazard ratio (95% CI)	HR *P* value	Log-rank *P* value

Receptor status				
ER/PR+^#^	73 (77.66)	0.51 (0.06–4.19)	0.530	0.732
Her2+	8 (8.51)	0.49 (0.01–19.06)	0.706	
Triple negative	13 (13.83)	—		
Grade				
1	26 (26)	0.20 (0.01–6.55)	0.370	0.339
2	42 (42)	1.08 (0.17–6.88)	0.935	
3	32 (32)	—		
Surgery type				
Lumpectomy	92 (87.6)	0.06 (0.01–0.36)	0.002	<0.001
Mastectomy	13 (12.4)	—		
Adjuvant chemotherapy				
No	95 (90.48)	0.33 (0.04–2.50)	0.282	0.447
Yes	10 (9.52)	—		
Neoadjuvant chemotherapy				
No	98 (93.33)	0.03 (0.00–0.27)	0.001	<0.001
Yes	7 (6.67)	—		
Detected on mammography				
No	38 (36.89)	6.44 (0.84–49.11)	0.073	0.022
Yes	65 (63.11)	—		
Stage at diagnosis				
0/I	68 (65.38)	0.34 (0.06–2.02)	0.237	0.187
II/III	36 (34.62)	—		
Nodal status				
Positive	24 (22.86)	4.77 (0.81–28.25)	0.085	0.046
Negative	81 (77.14)	—		
Postmastectomy radiation				
No	3 (23.08)	2.96 (0.27–32.47)	0.374	0.454
Yes	10 (76.92)	—		
Postlumpectomy radiation				
No	7 (7.61)	3.42 (0.08–141.16)	0.517	0.733
Yes	85 (92.39)	—		
Final margin status				
Positive	2 (1.96)	4.19 (0.15–115.22)	0.397	0.549
Less than 2 mm	21 (20.59)	0.42 (0.02–11.35)	0.603	
Greater than 2 mm	79 (77.45)	—		

^*^Total patients for each characteristic may vary due to incomplete medical records for some patients.

^#^ER/PR+: estrogen receptor/progesterone receptor positive.
